# The Interactive Effects of the Anti-Sea Lice Pesticide Azamethiphos and Temperature on Oxidative Damage and Antioxidant Responses in the Oyster *Ostrea chilensis*

**DOI:** 10.3390/antiox13060737

**Published:** 2024-06-17

**Authors:** Jaime A. Montory, Victor M. Cubillos, Oscar R. Chaparro, Paulina Gebauer, Matthew R. Lee, Eduardo Ramírez-Kuschel, Francisco Paredes-Molina, Valentina Lara-Sandoval, Juan P. Cumillaf, Luis P. Salas-Yanquin, Joseline A. Büchner-Miranda

**Affiliations:** 1 Centro i~mar, Universidad de los Lagos, Casilla 557, Puerto Montt 5480000, Chile; pgebauer@ulagos.cl (P.G.); matthew.lee@ulagos.cl (M.R.L.); valentinasansan@gmail.com (V.L.-S.); 2 Instituto de Ciencias Marinas y Limnológicas, Universidad Austral de Chile, Valdivia 5090000, Chile; victor.cubillos@uach.cl (V.M.C.); ochaparr@uach.cl (O.R.C.); edrramirez@aim.com (E.R.-K.); paredesfco91@gmail.com (F.P.-M.); joseline.a.buchner@gmail.com (J.A.B.-M.); 3 Programa de Doctorado en Ciencias de la Acuicultura, Universidad Austral de Chile, Los Pinos s/n, Balneario Pelluco, Puerto Montt 5480000, Chile; jpnenen@gmail.com; 4 Facultad de Ciencias, Unidad Multidisciplinaria de Docencia e Investigación, Universidad Nacional Autónoma de México, Puerto de Abrigo s/n, Sisal 97356, Mexico; luis.salas.y@gmail.com

**Keywords:** Chilean oyster, Bivalvia, azamethiphos, temperature, oxidative damage, antioxidant response

## Abstract

Azamethiphos is used in the salmon industry to treat sea lice and is subsequently discharged into the sea, which may affect non-target species (NTS). A rise in seawater temperature could enhance the sensitivity of NTS. Thus, in the present investigation, the combined effects of azamethiphos (0 µg L^−1^, 15 µg L^−1^ and 100 µg L^−1^) and temperature (12 °C and 15 °C) was assessed over time (7 days) in the gonads and gills of the oyster *Ostrea chilensis*, assessing its oxidative damage (lipid peroxidation and protein carbonyls) and total antioxidant capacity. Our results indicated that in gonads and gills, lipid peroxidation levels increased over time during exposure to both pesticide concentrations. Protein carbonyl levels in gills increased significantly in all experimental treatments; however, in gonads, only pesticide concentration and exposure time effected a significant increase in protein damage. In both, gill and gonad temperature did not influence oxidative damage levels. Total antioxidant capacity in gonads was influenced only by temperature treatment, whereas in the gills, neither temperature nor azamethiphos concentration influenced defensive responses. In conclusion, our results indicated the time of pesticide exposure (both concentrations) had a greater influence than temperature on the cellular damage in this oyster.

## 1. Introduction

Salmon aquaculture has been growing steadily across the world over recent decades [1], particularly in the Southern Hemisphere, where salmon and trout are intensively farmed in the fjords and channels of Chilean Patagonia [2,3]. Due to the high fish densities utilized in commercial salmon farming, the fish are susceptible to parasitic diseases, such as sea lice, requiring the use of chemical treatments [4]. In Chile, the predominant species of sea lice is the ectoparasitic copepod *Caligus rogercresseyi* [2,5,6,7,8]. Chemical pesticides are the most commonly utilized form of antiparasitic treatment and are routinely used against sea lice in salmon aquaculture [1,3]. Different types of chemical antiparasitic pesticides have been used to control sea lice, including those formulated with organophosphates, pyrethroids, or hydrogen peroxide [3,9]. Currently, the organophosphate antiparasitic azamethiphos (Salmosans^®^) is one of the most effective chemical treatments against sea lice and is used in several countries of the European Union according to the EC Regulation 1107/2009 [10] and in Chile [1,2,3,4,11]. Azamethiphos is applied using baths with a recommended concentration of 100 μg L^−1^ for 1 h of exposure [9]. After completion of the chemical treatment, the compound is discharged into the surrounding seawater. Thus, large volumes of azamethiphos pesticide are discharged into the Chilean marine coastal ecosystem [1], creating a potential problem for non-target species due to repeated low concentration exposures. Azamethiphos is characterized by being soluble in water and persisting in the marine environment for up to nine days [12]. Concentrations between 1 and 25 µg L^−1^ have been measured from 1 to 1000 m away from the application area [12]. Azamethiphos treatments are applied repeatedly to attack all parasitic stages of the sea lice lifecycle, using the pesticide in multiple pens within one farm, as well as in multiple farms within a single area, resulting in non-target species being subjected to repeated pulses of this pesticide [9,13,14]. Negative impacts of the pesticide azamethiphos on the survival, physiology and behavior of non-target species have been described in several species of marine invertebrates (*Metacarcinus edwardsii* [2]; *Ostrea chilensis* [11]; *Crangon septemspinosa* and *Mysis stenolepsis* [12]; *Homarus americanus* [15]; *Mytilus edulis* [16]; and *Padalus borealis* [17]). However, at the biochemical level, few studies have recorded the impact of the pesticide on non-target species [18]. For example, damage by oxidative stress through protein carbonylation was reported for *Homarus americanus* as a result of chronic exposure to 0.061 µg L^−1^ azamethiphos, indicating that azamethiphos produces oxidative stress [18]. In a study undertaken by [19], Glutathione S-Transferase (GST) inductions in crab *Carcinus maenas* required short exposure times (24 h) to assess the effects against exposure to chemical pesticides. Also, in the oyster *Crassostrea gigas*, the exposure of juveniles to pesticides produced significant effects on the level of lipid peroxidation and the activities of enzymes involved in oxidative stress defenses and detoxification [20,21,22]. It has been mentioned that many invertebrate responses to toxic compounds are determined by environmental factors (e.g., temperature), which could affect responses at the biochemical level [23]. Ref. [23] demonstrated strong interactions between the pesticide deltamethrin and seawater temperature on antioxidant enzyme activity in the black tiger shrimp *Penaeus monodon*, indicating that sensitivity to the pesticide could be influenced by the seawater temperature.

Bivalves such as oysters are considered ideal bio-indicators because of their sedentary lifestyle and sensitivity to environmental pollutants (sub-lethal effects) [24]. In Chile, the bivalve mollusk *Ostrea chilensis* (Chilean oyster) is of commercial importance with cultivated and natural populations occurring near salmon aquaculture sites. The oyster *O. chilensis* is distributed in southern Chile from Chiloé to the Guaitecas Islands [25,26] and is cultivated principally in the northern zone of Chiloé Island, Region de Los Lagos [27,28]. Currently, in the Region, more than 225 tons of *O. chilensis* are harvested yearly [28]. *O. chilensis* broods its embryos in the mantle cavity for 8 weeks [26]. Then, the females of this species release large and well-developed pediveliger larvae with an extremely short pelagic phase of only a few minutes to 48 h. *O. chilensis* females brood a high number of offspring (embryos per female: 3200–113,000) [27,28]. Thus, considering the ecotoxicological effect of the pesticide previously described, the objective of this research was to identify the interactive effects of azamethiphos and environmental temperature on the levels of oxidative damage and the total antioxidant response in adult individuals of the bivalve *O. chilensis.*

## 2. Materials and Methods

### 2.1. Collection and Maintenance of O. chilensis

Individuals of *O. chilensis* used for this study (shell length 50–60 mm) were obtained from the Quempillén estuary, the Island of Chiloé, southern Chile (41°52′ S, 73°46′ W; N total = 600). They were then immediately transferred to the Coastal Laboratory of Aquatic Resources, Universidad Austral de Chile, Valdivia, Chile. The experimental animals were kept for 10 days in laboratory aquaria supplied with filtered seawater (12 °C and salinity of 32 psu) and constant aeration. Seawater was changed every other day and the oysters were fed with a monoculture of the microalgae *Isochrysis galbana* (30,000 cel mL^−1^).

### 2.2. Experimental Design

To evaluate the interactive effects of temperature and azamethiphos exposure on *O. chilensis,* the following experimental design was employed. Two large thermoregulated water aquaria were used, the first at 12 °C (control), which corresponded to the average seawater temperature of the Quempillén estuary where the oysters were collected. The second was at 15 °C, three degrees higher than at the sampling site, considering the increase in seawater temperature predicted for the end of the century [29].

Three experimental azamethiphos (Salmosan^®^ Vet) treatments were used; 0 µg L^−1^ (control, without pesticide), 15 µg L^−1^ and 100 µg L^−1^. The 15 µg L^−1^ treatment represented the average concentration of azamethiphos recorded in the water column four hours after the application of the treatment in a salmon farm [12]. The 100 µg L^−1^ treatment is the recommended concentration for the treatment of sea lice [9]. According to [30], the linear hydrolytic decomposition of azamethiphos is 1% degradation at 3 h and 21% at 3 days. Therefore, in the case of our experiments, the hydrolysis decomposition of azamethiphos of 0.33% was estimated at 1 h of daily exposure [31], which was represented by the concentrations used (15 and 100 μg L^−1^).

Each treatment was replicated four times at each of the experimental temperatures. Each replicate consisted of a 15 L glass aquaria with a salinity of 32 psu, constant aeration and filtered seawater (0.5 µm) placed in the large thermoregulated aquaria (12 or 15 °C). Each aquaria contained 16 oysters. Two different responses of the oysters to the treatments were measured: oxidative damage (lipid peroxidation and protein carbonyl) and total antioxidant capacity (TAC).

**Figure 1:**
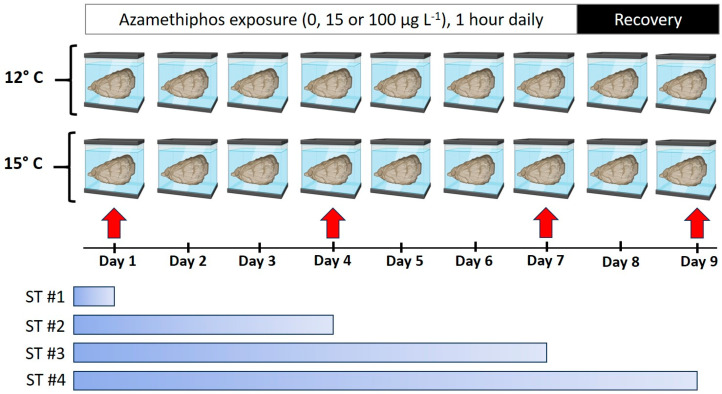
Experimental design of the four sampling times (ST 1–4) of *O. chilensis* during combined exposure to azamethiphos (0 (control),15 and 100 µg L^−1^, 1 h daily for 7 consecutive days) and temperature (12 and 15 °C) at days 1, 4 and 7. Days 8 and 9 correspond to the recovery period, without exposure to the pesticide.

All the oysters were kept in an open system with circulating seawater (dripping seawater) without azamethiphos, with constant aeration and being fed daily (microalgae *Isochrysis galbana*), and in one of the two temperature treatments (12 or 15 °C). Then, each day, oysters were placed in separate aquaria, with one of the azamethiphos treatments, and maintained at the appropriate temperature (12 or 15 °C). Thus, the oysters were exposed to the combined treatments of pesticide (0 control, 15 or 100 μg L^−1^) and temperature (12 or 15 °C) for 1 h (Figure 1), after which the oysters were returned to their original aquariums without azamethiphos. The procedure was repeated for seven consecutive days, following the procedure indicated by the Chilean National Fisheries Service. 

### 2.3. Oxidative Damage

#### 2.3.1. Lipid Peroxidation Levels

Lipid peroxidation (LP) levels were determined by quantifying using the malondialdehyde concentration (MDA) protocol [32]. Samples of gill and gonad were taken from 4 oysters in each treatment combination after 1, 4 and 7 days of exposure to the treatments and 2 days after the final exposure (day 9) (Figure 1). The samples were snap frozen with liquid nitrogen and were stored at −80 °C. Each of the samples (gills and gonads separately) were then analyzed as follows: 30 mg of pulverized frozen tissue was homogenized with 500 μL of trichloroacetic acid (TCA) (0.1% *w*/*v*) and centrifuged for 10 min at 13,000 rpm at 4 °C. Then, a 200 μL aliquot of the supernatant was homogenized with 500 μL of a solution of thiobarbituric acid (TBA) (0.5% *w*/*v*) and TCA (20% *w*/*v*) and heated for 30 min at 80 °C using a thermomixer (Eppendorf, Hamburg, Reinbek, Germany). Then, the samples were immediately placed on ice for 5 min and were then centrifuged for 5 min at 4 °C and at 13,000 rpm. The absorbance of the supernatant was determined at 520 nm using a microplate reader (Zenyth 200, Anthos, Biochrom Ltd., Cambridge, UK) and the LP in gonads and gills were expressed in nmol MDA g FW^−1^.

#### 2.3.2. Protein Carbonyl Levels

Protein oxidation levels were estimated by quantifying protein carbonyls, which was determined using the dinitrophenylhydrazine (DNPH) protocol [33]. Samples of gills and gonads were taken from 4 oysters in each treatment combination after 1, 4 and 7 days of exposure to the treatments and 2 days after the final exposure (day 9) (Figure 1). The samples were snap frozen separately using liquid nitrogen and were stored at −80 °C. Each of the samples (gills and gonads separately) were then analyzed as follows: 80 mg of frozen tissue, previously ground, was homogenized with liquid nitrogen and saline extraction buffer (1% polyvinylpyrrolidone (PVP) and 0.1 mM ethylenediaminetetraacetic acid (EDTA)) and centrifuged at 4 °C for 20 min (13,500 rpm). Proteins from the extract were precipitated with 200 μL TCA 20% and frozen for 30 min at −20 °C and subsequently separated from the homogenate by centrifugation (13,500 rpm) at 4 °C for 10 min. The supernatant was discarded, and the pellet was re-suspended with 300 μL of 100 mM DNPH in 2 N HCl and incubated at room temperature for 1 h. Then, 500 μL of TCA 20% was added, and the sample was frozen again at −20 °C for 15 min. The solution was centrifuged (13,500 rpm) for 10 min at 4 °C and the supernatant was eliminated. The pellet was re-suspended with a solution of 500 μL of ethanol/ethyl acetate (1:1) and then centrifuged (14,000 rpm) at 4 °C for 10 min. The supernatant was discarded again, and the resulting pellet was re-suspended with 1 mL of guanidine HCl 6 M and centrifuged (14,000 rpm) at 4 °C for 20 min. Finally, the absorbance of the supernatant was determined at 380 nm using a plate reader (Zenyth 200, Anthos, Biochrom Ltd.).

A soluble protein extract was generated for the determination of protein carbonyls, for which 1% PVP and 1 mL of 1X phosphate-buffered saline (PBS) buffer pH 7.4 100 mM EDTA were added to 100 mg of frozen ground tissue and centrifuged for 20 min (14,000 rpm). The total concentration of the protein extract was determined according to the specifications of the commercial protein determination kit (Pierce BCA, Abcam Company, Waltham, MA, USA). After the concentrations were obtained, the concentration of all samples was equalized to a minimum of 200 μg protein per mg frozen weight. Then, an aliquot of 200 μL of protein extract was used to determine protein carbonyl levels in gills and gonads that were expressed in μmol carbonyl mg protein^−1^.

### 2.4. Total Antioxidant Capacity

Levels of total antioxidant capacity (TAC) were determined using the protocol of DPPH [34] in the gills and gonads of *O. chilensis* after 1, 4 and 7 days of exposure to the treatments and 2 days after the final exposure to the azamethiphos treatments (9 day) (Figure 1). TAC was determined by homogenizing 60 mg of frozen tissue (gills and gonads separately), previously ground with liquid nitrogen, in 1 mL of 70% acetone, mixing well and sonicating in an ultrasonic bath for 2 h at 4 °C. The samples were then centrifuged for 10 min at 7500 rpm (4 °C). The supernatant was reserved in a new tube and placed under a laboratory fume hood to allow the acetone to evaporate to a volume of 500 μL. Then, a 200 μL volume of 2,2-diphenyl-1-picrylhydrazyl (DPPH) (150 μM 70% acetone) was placed in each well of a 96-well microplate, and 25 μL of the extract of each sample was added, and then the absorbance was determined at 520 nm through a kinetic loop for 2 h (37 °C) using a Zenith 200 microplate reader (Anthos, Biochrom Ltd.). Levels of total antioxidant capacity were estimated using Trolox (synthetic antioxidant/6-Hydroxy-2,5,7,8-tetramethylchromane-2-carboxylic acid), with the values expressed in mg Trolox Eq g FW^−1^.

### 2.5. Statistical Analysis

After testing for normality (the Shapiro–Wilk test) and homoscedasticity (the Levene test), three-way ANOVA was used to evaluate the effect of temperature, azamethiphos concentration and exposure time on the levels the lipid peroxidation, protein carbonylation and total antioxidant capacity of *O. chilensis*. A posteriori Tukey tests were used when significant differences were detected. We used a significance level of 0.05 for each analysis [35]. All data were analyzed using the software Statistica V.7.

## 3. Results

### 3.1. Lipid Peroxidation Levels

Lipid peroxidation levels in the gonads of *O. chilensis*, varied significantly with exposure time (F_(3,72)_ = 47.62; *p* = 0.0001; Table 1(A)), the concentration of the azamethiphos (F_(2,72)_ = 19.40; *p* = 0.0001; Table 1(A)) and in the interaction between exposure time and the azamethiphos concentration (F_(6,72)_ = 9.59; *p* = 0.0001; Table 1(A)). However, no significant differences were identified between temperatures (F_(1,72)_ = 3.63; *p* = 0.0605; Table 1(A)), neither in the interactions of temperature and exposure time (F_(3,72)_ = 1.96; *p* = 0.1264; Table 1(A)) and temperature and azamethiphos concentration (F_(2,72)_ = 2.48; *p* = 0.0907; Table 1(A)) nor in the interaction between exposure time, temperature and azamethiphos concentration (F_(6,72)_ = 0.45; *p* = 0.8376; Table 1(A)).

**Figure 2:**
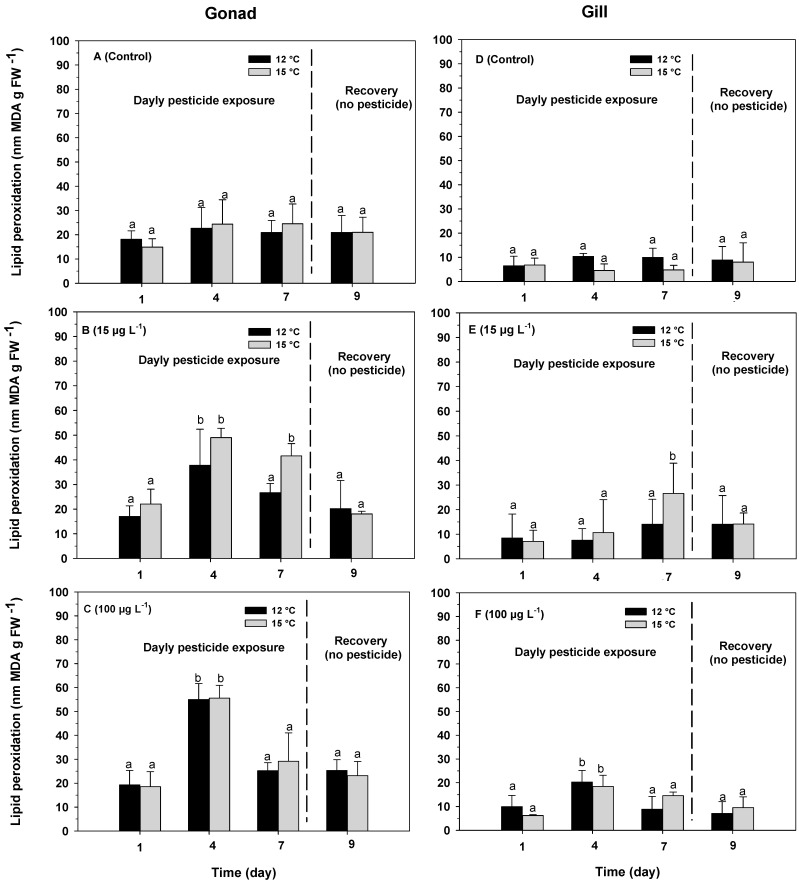
Lipid peroxidation levels in gonads (**A**–**C**) and gills (**D**–**F**) in adults of the oyster *O. chilensis* exposed to temperature of 12 °C (black bars) and 15 °C (gray bars) and azamethiphos concentrations of 0 ((**A**,**D**); without pesticide, control), 15 µg L^−1^ (**B**,**D**) and 100 µg L^−1^ (**C**,**F**) in relation to exposure time (1, 4 and 7 days of exposure and 2 days of recovery). Error bars indicate SD. Different letters indicate significant differences.

Lipid peroxidation levels in the gonad remained constant over time in the control treatments, with an average value of 20.67 ± 6.01 and 21.21 ± 6.95 nm MDA g FW^−1^ at temperatures of 12 and 15 °C, respectively (Figure 2A). During the period of daily exposure to azamethiphos, the highest levels of lipid peroxidation were recorded after 4 days in individuals exposed to concentrations of 15 µg L^−1^ and 100 µg L^−1^, reaching values of 37.76 ± 14.68 and 49.02 ± 3.80 nm MDA g FW^−1^ at temperature of 12 °C and 55.01 ± 6.71 and 55.58 ± 5.42 nm MDA g FW^−1^ at a temperature of 15 °C, respectively (Figure 2B,C). After exposure to azamethiphos ceased (day 9), in both temperature treatments and concentrations, the lipid peroxidation levels decreased significantly, reaching the same levels as those registered in the control treatments (Figure 2B,C).

Lipid peroxidation levels in the gills of *O. chilensis* varied significantly with exposure time (F_(3,72)_ = 3.74; *p* = 0.0146; Table 2(A)), the concentration of azamethiphos (F_(2,72)_ = 5.53; *p* = 0.0058; Table 2(A)) and in the interaction between exposure time and azamethiphos concentration (F_(6,72)_ = 3.11; *p* = 0.0090; Table 2(A)). However, no significant differences were observed between temperature treatments (F_(1,72)_ = 0.41; *p* = 0.5221; Table 2(A)) nor in the interactions between temperature and exposure time (F_(3,72)_ = 0.27; *p* = 0.8562; Table 2(A)), temperature and azamethiphos concentration (F_(2,72)_ = 0.86; *p* = 0.4236; Table 2(A)), or exposure time, temperature and azamethiphos concentration (F_(6,72)_ = 1.27; *p* = 0.2814; Table 2(A)). Lipid peroxidation levels remained constant over time in the control treatments, with an average value of 7.44 ± 3.78 nm MDA g FW^−1^ (Figure 2D). For the individuals exposed to the 15 µg L^−1^ treatment, the highest levels of lipid peroxidation were recorded after 7 days of exposure to azamethiphos, with values of 14.05 ± 10.17 and 26.57 ± 12.37 nm MDA g FW^−1^ for the temperature treatments of 12 and 15 °C, respectively (Figure 2E). After exposure to azamethiphos ceased (day 9), the lipid peroxidation levels decreased, reaching the values registered in the control treatments at both temperatures (Figure 2E). For the individuals exposed to the 100 µg L^−1^ treatment, at both temperatures, the highest levels of lipid peroxidation were recorded after 4 days of exposure to azamethiphos, reaching values of 20.32 ± 4.88 and 18.44 ± 4.70 nm MDA g FW^−1^ for the temperature treatments of 12 and 15 °C, respectively (Figure 2F). After exposure to azamethiphos ceased (day 9), lipid peroxidation levels decreased, reaching baseline values as observed in the control treatment at both temperatures and concentrations (Figure 2E,F).

### 3.2. Protein Carbonyl Levels

**Figure 3:**
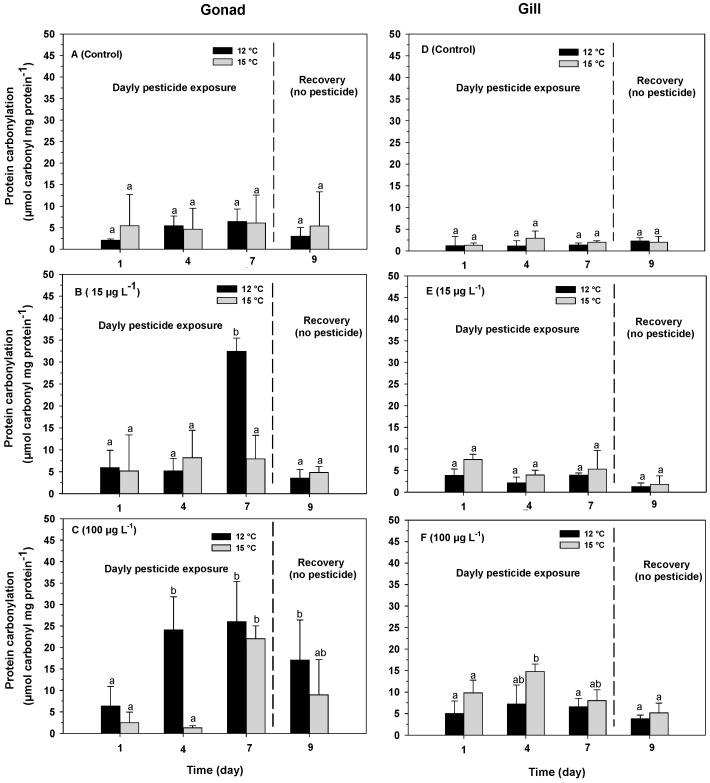
Protein carbonyl levels in gonads (**A**–**C**) and gills (**D**–**F**) in adults of the oyster *O. chilensis* exposed to temperature of 12 °C (black bars) and 15 °C (gray bars) and azamethiphos concentration of 0 ((**A**,**D**); without pesticide, control), 15 µg L^−1^ (**B**,**D**) and 100 µg L^−1^ (**C**,**F**) in relation to exposure time (1, 4 and 7 days of exposure and 2 days of recovery). Error bars indicate SD. Different letters indicate significant differences.

Protein carbonyl levels in the gonads of *O. chilensis* varied significantly between exposure times (F_(3,72)_ = 26.93; *p* = 0.0001; Table 1(B)), azamethiphos concentration (F_(2,72)_ = 19.00; *p* = 0.0001; Table 1(B)) and temperature (F_(1,72)_ = 12.05; *p* = 0.0008; Table 1(B)). In addition, significant differences were observed in the interactions between temperature and azamethiphos (F_(2,72)_ = 7.24; *p* = 0.0013; Table 1(B)), exposure time and azamethiphos (F_(6,72)_ = 6.20; *p* = 0.0001; Table 1(B)) and the interaction between exposure time, azamethiphos and temperature (F_(6,72)_ = 4.02; *p* = 0.0015; Table 1(B)). However, no significant differences were observed in the interaction between temperature and exposure time (F_(3,72)_ = 2.15; *p* = 0.1005; Table 1(B)). Protein carbonyl levels remained constant over time in the control treatments, registering average values of 4.23 ± 1.89 and 5.39 ± 6.64 µmol carbonyl mg protein^−1^ at 12 and 15 °C, respectively (Figure 3A). Oysters exposed to an azamethiphos concentration of 15 µg L^−1^ and kept at 12 °C showed a significant increase in protein carbonyl levels after 7 days of exposure, with a value of 32.43 ± 5.39 µmol carbonyl mg protein^−1^, which was four times higher than that of the individuals kept at 15 °C (Figure 3B). Once the exposure to azamethiphos ceased, in the individuals kept at 12 °C, the levels of protein carbonyls decreased to values of 3.52 ± 2.04 µmol carbonyl mg protein^−1^ (Figure 3B). In oysters exposed to an azamethiphos concentration of 100 µg L^−1^, in both temperature treatments, protein carbonyl levels reached their maximum values after 7 days of exposure, registering values of 26.01 ± 9.39 and 22.04 ± 3.01 µmol carbonyl mg protein^−1^ at 12 and 15 °C, respectively (Figure 3C). Once the exposure to azamethiphos ceased, protein carbonyl levels decreased in both temperature treatments, registering an average value of 13.01 ± 8.79 µmol carbonyl mg protein^−1^, but were higher than the control (Figure 3C).

Protein carbonyl levels in the gills of *O. chilensis*, varied significantly between temperature (F_(1,72)_ = 25.31; *p* = 0.0003; Table 2(B)), exposure time (F_(3, 72)_ = 5.53; *p* = 0.0002; Table 2(B)) and azamethiphos concentration (F_(2,72)_ = 67.75; *p* = 0.0001; Table 2(B)). In addition, there were also significant differences in the interactions between temperature and exposure time (F_(3,72)_ = 3.11; *p* = 0.0313; Table 2(B)), temperature and azamethiphos concentration (F_(2,72)_ = 5.16 ; *p* = 0.0080; Table 2(B)) and exposure time and azamethiphos concentration (F_(6,72)_ = 6.52; *p* = 0.0001; Table 2(B)). However, no significant differences were observed in the interaction between the exposure time, temperature and azamethiphos concentration (F_(6,72)_ = 1.21; *p* = 0.3095; Table 2(B)). The protein carbonyl levels did not vary in the controls at either temperature (Figure 3D). In the oysters exposed to an azamethiphos concentration of 15 µg L^−1^, the gills registered average values of 2.80 ± 1.05 and 4.66 ± 1.61 µmol carbonyl mg protein^−1^ at 12 and 15 °C, respectively (Figure 3E). For oysters exposed to an azamethiphos concentration of 100 µg L^−1^, at both temperatures, maximum protein carbonyl levels were recorded after 4 days, averaging 7.22 ± 4.43 and 14.78 ± 1.74 µmol carbonyl mg protein^−1^ at 12 and 15 °C, respectively (Figure 2F). Once the exposure to azamethiphos ceased, at both temperatures, the protein carbonyl levels returned to the initial levels, registering values of 3.81 ± 0.83 and 5.18 ± 2.25 µmol carbonyl mg protein^−1^ at 12 and 15 °C, respectively (Figure 3E,F).

### 3.3. Total Antioxidant Capacity

**Figure 4:**
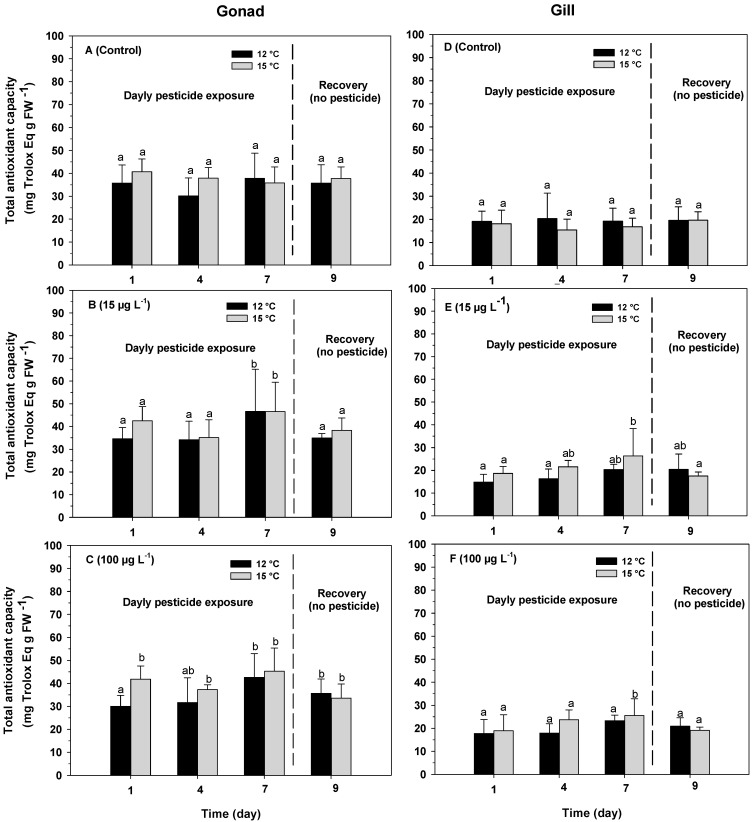
Total antioxidant capacity in gonads (**A**–**C**) and gills (**D**–**F**) in adults of the oyster *O. chilensis* exposed to temperature of 12 °C (black bars) and 15 °C (gray bars) and azamethiphos concentration of 0 ((**A**,**D**); without pesticide, control), 15 µg L^−1^ (**B**,**D**) and 100 µg L^−1^ (**C**,**F**) in relation to exposure time (1, 4 and 7 days of exposure and 2 days of recovery). Error bars indicate SD. Different letters indicate significant differences.

Total antioxidant capacity in the gonads of *O. chilensis* varied significantly between temperatures (F_(1,72)_ = 4.51; *p* = 0.0369; Table 1(C)) and exposure time (F_(3,72)_ = 4.27; *p* = 0.0078; Table 1(C)). However, no differences were observed between the azamethiphos treatments (F_(2,72)_ = 0.83; *p* = 0.4367; Table 1(C)) nor in any of the possible interactions between the temperature, exposure time and azamethiphos concentration (Table 1(C)). The oysters belonging to the control treatment and those that were exposed to an azamethiphos concentration of 15 µg L^−1^ did not vary significantly in the total antioxidant capacity over the exposure period, presenting an average value of 36.45 ± 7.11 and 39.05 ± 8.27 mg Trolox Eq g FW^−1^ at 12 and 15 °C, respectively (Figure 4A,B). Oysters exposed to an azamethiphos concentration of 100 µg L^−1^ presented the highest values of total antioxidant capacity, after 7 days of exposure, registering an average of 42.58 ± 10.37 and 45.27 ± 10.07 mg Trolox Eq g FW^−1^ at 12 and 15 °C, respectively (Figure 4C), subsequently decreasing to 34.61 ± 6.19 mg Trolox Eq g FW^−1^ at both temperatures after exposure to azamethiphos had ceased (Figure 4C).

Total antioxidant capacity in the gills of *O. Chilensis* varied significantly only with exposure time (F_(3,72)_ = 3.06; *p* = 0.0334; Table 2(C)). There were no significant differences with temperature (F_(1,72)_ = 1.25; *p* = 0.2660; Table 2(C)) nor azamethiphos concentration (F_(2,72)_ = 2.25; *p* = 0.1123; Table 2(C)). In addition, no significant differences were identified in any of the possible interactions between temperature, exposure time and azamethiphos concentration (Table 2(C)). The total antioxidant response did not vary between the controls (without pesticide) nor with azamethiphos concentrations of 15 and 100 µg L^−1^, registering values of 18.50 ± 5.61, 19.52 ± 4.53 and 20.92 ± 4.49 mg Trolox Eq g FW^−1^, respectively (Figure 4D–F).

## 4. Discussion

Rising environmental temperatures and the use of chemical pesticides are two stress factors that each threaten global biodiversity [36], and the interaction between these two stressors has the potential for additive or synergistic negative impacts on organisms [36,37,38]. The increases in environmental temperature have the potential to change the level of toxicity of a pesticide in the environment [38,39]. This interaction between temperature and pesticides may be one of the underlying reasons why pesticides concentrations that current legislation considers safe are causing biodiversity decline in aquatic ecosystems [38,40,41].

The interactive effects between pesticides commonly used in aquaculture and variations in environmental temperature not only have negative impacts on the physiology and survival of non-target aquatic invertebrate species [11,15,16], but they also have impacts on the biochemical parameters at the cellular level [24,42,43,44,45]. In the present study, the interaction between environmental temperature and the pesticide azamethiphos caused a significant increase in the levels of lipid peroxidation and protein carbonyls in *O. chilensis*. Similar results of oxidative stress have been identified in other species of aquatic invertebrates exposed to the interaction of environmental temperature and chemical pollutants (*Penaeus monodon* [23]; *Bellamya bengalensis* [42]; *Unio tumidus* [43]; and *Nacella concinna* [45]). Organic xenobiotics are pro-oxidant chemicals that can generate different pathways of oxidative challenges at cellular and molecular levels, generating a complex network of interactions and cascade effects in those organisms that assimilate them [46]. Pollutant-induced ROS are formed though transcriptional and post-transcriptional mechanisms contributing to inducing oxidative damage [46]. Particularly, it has been described that an organic xenobiotic induces intracellular ROS through the transcription of the cytochrome P450 multigene family, which catalyzes a variety of oxidative reactions (hydroxylation, epoxidation, dealkylation, deamination, sulfoxidation and desulfuration) to finally generate a peroxide [46,47]. Additionally, xenobiotics can generate the release of transition metals such as Fe and Cu from peroxisomes, a situation that catalyzes the generation of HO^●^ through Fenton reaction [48]. Thus, the presence of xenobiotics such as azamethiphos in the aqueous media can trigger elevated levels of ROS as pro-oxidant mechanisms affecting the cellular viability of *O. chilensis* as was observed in this experimental approach.

Additionally, it is possible that increases in oxidative stress levels identified in our results could be related to an increase in the oxygen consumption rate in *O. chilensis* exposed to the same interactive experimental treatments of environmental temperature and azamethiphos pesticide [11]. During exposure to these environmental stressors, there was an increase in the metabolic rate (oxygen consumption), which led to an increase in the release of reactive oxygen species in the electron transport chain [49,50,51]. This can be exacerbated by the interaction between chemical stressors and higher environmental temperature, increasing the risk of oxidative damage in organisms [52]. An increase in oxidative stress levels is critical for the integrity of cell membranes, which can lead to an alteration of subcellular structures and general cellular homeostasis [50]. This leads to oxidative damage, which promotes cell death and eventually limits the survival of organisms under stressful conditions [51,52,53].

Antioxidant enzymes convert reactive oxygen species (ROS) into stable non-toxic molecules, rendering them harmless, and are therefore the most important defense mechanism against cell damage induced by oxidative stress [53,54]. Despite temperature and pesticide exposure being correlated with an increase in enzymatic activity and physiological processes [46,55], in our results, an increase in reactive oxygen species (oxidative stress) but not in total antioxidant capacity was observed. Chemical contaminants cause alterations in the metabolism of an organism [11,56,57,58], which could impact its capacity to activate defense mechanisms, such as antioxidant enzymes, enhancing possible cell damage by oxidative stress [58,59]. When bivalve mollusks are exposed to contaminants, the production of ROS generally increases, leading to the activation of antioxidant defenses [60]. However, our results indicate that at neither of the two experimental temperatures (12 and 15 °C) did the activity of antioxidant defenses increase during exposure to the pesticide azamethiphos. This may indicate that the concentrations and exposure times of the oysters were not enough to activate the antioxidant defense mechanisms or that other defense mechanisms (e.g., detoxification) could be sufficient to prevent cell damage, especially at the highest concentrations of azamethiphos [59]. In *O. chilensis*, the interaction between the pesticide azamethiphos and environmental temperature led to an increase in metabolic rates [11], which accelerated anabolic and catabolic processes, including detoxification mechanisms and the excretion of xenobiotics, which may have been accompanied by an increase in immunological defenses [52]. Moreover, between the cellular responses to temperature increases and environmental pollution are the expression of heat shock proteins (HSPs) and metallothioneins (MTs), which play a key role in cellular protection [61,62]. In this sense, it has been observed that stress proteins of the HSP70 type, generated during stress, are involved in the process of the detoxification of protein carbonates through processes of the proteolysis and proteosomal degradation of oxidized proteins [63]. For example, ref. [60] identified as cellular protective mechanisms high levels of HSP70 and MTs in the oyster *Crassostrea virginica* exposed to high temperatures and cadmium, which, in our results, could be related to the rapid recovery in the levels of lipid peroxidation and protein carbonyls without an increase in total antioxidant levels. Results similar to ours were reported by [64,65] in the bivalve mollusk *Mytilus galloprovincialis* exposed to the interactive treatments of temperature and chemical contaminants. Also, the investigations carried out by [51,66] recorded an increase in the levels of oxidative damage but not in antioxidant activity in the bivalves *Mytilus edulis* and *Mya arenaria*, when they were exposed to higher temperatures and chemical herbicides, respectively. Similarly, in the clam *Donax incarnatus,* an inhibition of the activity of antioxidant enzymes was also observed as a result of the exposure of the bivalve to the organophosphate pesticide monocrotophos [53].

In summary, in our results, the combination between the pesticide azamethiphos and an increase in ambient temperature caused increases in the concentrations of oxidative damage indicators, without an identified response of the antioxidant defense of *O. chilensis*, which could eventually lead to oxidative damage in organisms. This, in turn, could lead to a negative impact on fitness [67], something that should be closely evaluated in future research on *O. chilensis*.

The interactions of chemical pollutants with other environmental stress factors have been poorly studied despite their relevance in explaining the effects on life history traits observed in a variety of organisms [68,69,70]. Given the strong connection between oxidative damage and the negative impact on the fitness of organisms [67], the increase in the levels of oxidative stress indicators probably indicates a reduction in the fitness of organisms [70].

In southern Chile, the use of chemical pesticides (e.g., azamethiphos) in salmon farming centers has generated special attention in recent years due to its proximity to shellfish farming centers (impacts on non-target species). Therefore, it is necessary to continue conducting research investigating not only pesticide–environmental interactions (pH, salinity and temperature), but it is also necessary to evaluate the impact of the repeated application of these chemical pesticides over time and the possible synergistic effects when used in combination with other chemicals routinely used in the salmon farming industry (cypermethrin, deltamethrin and hydrogen peroxide). In conclusion, the chemical compound azamethiphos, commonly used for treating the ectoparasite *Caligus rogercressegi*, has detrimental effects at the cellular level (oxidative damage) of adult *O. chilensis* individuals and potentially on many other non-target species. Therefore, more collaboration and research are necessary to move towards a sustainable and competitive aquaculture industry.

## 5. Conclusions

The lipid peroxidation levels in gonads and gills increased over time with exposure to both pesticide concentrations (15 and 100 mg L^−1^). Protein carbonyl levels in the gills increased significantly in all experimental treatments. However, in the gonads, only the pesticide concentration and exposure time were related to a significant increase in protein damage. In both gills and gonads, temperature did not influence the levels of oxidative damage. Total antioxidant capacity in the gonads was influenced only by the temperature treatment, while in the gills, neither temperature nor pesticide concentration influenced oxidative damage levels. Thus, according to our results, pesticide exposure time (at both concentrations) influences cell damage in this oyster more than temperature. Therefore, the pesticide azamethiphos, due to its lack of specificity and broad spectrum of action, could potentially affect many other non-target species. Future research should evaluate the combined impact of more than one pharmaceutical compound (e.g., deltamethrin and azamethiphos) on non-target species as there is no coordination in the application of these pharmaceuticals between salmon farms.

## Figures and Tables

**Table 1:** Three-way ANOVA to evaluate the effects of temperature (12 and 15 °C), azamethiphos concentration (0 (control), 15 and 100 μg L^−1^) and exposure time (1, 4 and 7 days of exposure and 2 days of recovery) on lipid peroxidation (nmol MDA g FW^−1^), protein carbonyl (μmol carbonyl mg protein^−1^) and total antioxidant capacity (mg Trolox Eq g FW^−1^) in the gonads of *O. chilensis. p* values in bold indicate statistical significance.

Source	df	MS	F	*p*
**(A)** **Lipid peroxidation**				
Temperature (°C)	1	180.76	3.63	0.0605
Time (Day)	3	2367.01	47.62	**0.0001**
Azamethiphos (µg L^−1^)	2	964.21	19.40	**0.0001**
Temperature × Time	3	97.81	1.96	0.1264
Temperature × Azamethiphos	2	123.35	2.48	0.0907
Azamethiphos × Time	6	476.78	9.59	**0.0001**
Temperature × Azamethiphos × Time	6	22.71	0.45	0.8376
Error	72	49.70		
**(B)** **Protein carbonyl**				
Temperature (°C)	1	382.26	12.05	**0.0008**
Time (Day)	3	854.11	26.93	**0.0001**
Azamethiphos (µg L^−1^)	2	602.71	19.00	**0.0001**
Temperature × Time	3	68.39	2.15	0.1005
Temperature × Azamethiphos	2	229.69	7.24	**0.0013**
Azamethiphos × Time	6	196.73	6.20	**0.0001**
Temperature × Azamethiphos × Time	6	127.67	4.02	**0.0015**
Error	72	31.70		
**(C)** **Total antioxidant capacity**				
Temperature (°C)	1	308.00	4.51	**0.0369**
Time (Day)	3	291.20	4.27	**0.0078**
Azamethiphos (µg L^−1^)	2	57.10	0.83	0.4367
Temperature × Time	3	81.80	1.20	0.3160
Temperature × Azamethiphos	2	4.90	0.07	0.9302
Azamethiphos × Time	6	57.80	0.84	0.5378
Temperature × Azamethiphos × Time	6	23.60	0.34	0.9099
Error	72	68.20		

**Table 2:** Three-way ANOVA to evaluate the effects of temperature (12 and 15 °C), azamethiphos concentration (0 (control), 15 and 100 μg L^−1^) and exposure time (1, 4 and 7 days of exposure and 2 days of recovery) on lipid peroxidation (nmol MDA g FW^−1^), protein carbonyl (μmol carbonyl mg protein^−1^) and total antioxidant capacity (mg Trolox Eq g FW^−1^) in the gills of *O. chilensis. p* values in bold indicate statistical significance.

Source	df	MS	F	*p*
**(A)** **Lipid peroxidation**				
Temperature (°C)	1	13.40	0.41	0.5221
Time (Day)	3	121.47	3.74	**0.0146**
Azamethiphos (µg L^−1^)	2	179.22	5.53	**0.0058**
Temperature × Time	3	8.77	0.27	0.8562
Temperature × Azamethiphos	2	28.17	0.86	0.4236
Azamethiphos × Time	6	100.93	3.11	**0.0090**
Temperature × Azamethiphos × Time	6	41.19	1.27	0.2814
Error	72	32.40		
**(B)** **Protein carbonyl**				
Temperature (°C)	1	103.53	25.31	**0.0003**
Time (Day)	3	31.32	7.65	**0.0002**
Azamethiphos (µg L^−1^)	2	277.11	67.75	**0.0001**
Temperature × Time	3	12.74	3.11	**0.0313**
Temperature × Azamethiphos	2	21.12	5.16	**0.0080**
Azamethiphos × Time	6	26.68	6.52	**0.0001**
Temperature × Azamethiphos × Time	6	4.96	1.21	0.3095
Error	72	4.09		
**(C)** **Total antioxidant capacity**				
Temperature (°C)	1	39.27	1.25	0.2660
Time (Day)	3	95.75	3.06	**0.0334**
Azamethiphos (µg L^−1^)	2	70.46	2.25	0.1123
Temperature × Time	3	24.95	0.79	0.4988
Temperature × Azamethiphos	2	67.42	2.15	0.1230
Azamethiphos × Time	6	34.99	1.12	0.3596
Temperature × Azamethiphos × Time	6	23.70	0.75	0.6049
Error	72	31.26		

## Data Availability

The data presented in this study are available in a data repository.
